# Association between Food/UGT2B7 Polymorphisms and Pharmacokinetics/Pharmacodynamics Properties of Indapamide in Healthy Humans

**DOI:** 10.3390/biomedicines11051501

**Published:** 2023-05-22

**Authors:** Banaz Abbas, Nagwa A. Sabri, Amal A. El-Khouly

**Affiliations:** Department of Clinical Pharmacy, Faculty of Pharmacy, Ain Shams University, Cairo 4393005, Egypt; banazdara2017@yahoo.com (B.A.); amal.elkhouly@pharma.asu.edu.eg (A.A.E.-K.)

**Keywords:** indapamide, hypertension, food, pharmacokinetics, *UGT2B7*, polymorphism, pharmacodynamics, pharmacogenomics

## Abstract

Indapamide is an effective and safe antihypertensive medication showing a beneficial effect in combination with other antihypertensive agents regarding morbidity and mortality. A comparative study was performed under fasting and fed conditions to investigate the effect of food and selected single nucleotide polymorphisms in the uridine diphosphate glucuronyl transferase (*UGT2B7*) gene on the pharmacokinetics and pharmacodynamics behavior of indapamide 1.5 mg sustained release. Forty-nine healthy volunteers aged 18–55 years were randomized into two groups; 25 volunteers were administered indapamide under fasting conditions and 24 under fed conditions. Genotyping of the *UGT2B7 rs7438135* and *rs11740316* was done before commencing the study using predesigned TaqMan assays. Results showed that food independently decreased the value of indapamide’ T_max_ by 5.5 h and increased the value of C_max_ by 8.7 ng/mL. On the other hand, all genetic variants of both *UGT2B7* SNPs had no significant impact on the values of T_max_, C_max_, and AUC_0–t_; however, it was found that rs11740316 variant *AG* was correlated with a 2.8 h lower MRT_inf_. Finally, BMI positively correlated with longer MRT_inf_. It was concluded that none of *rs7438135*, *rs11740316*, or food had a significant impact on the pharmacodynamic properties. Food had a modest impact on indapamide C_max_ and T_max_ values, while there were unremarkable differences in safety and efficacy.

## 1. Introduction

Hypertension (HTN) is a prevalent chronic medical condition that is identified by a persistent elevation in arterial pressure. The current diagnostic criterion for hypertension is characterized by systolic blood pressure (SBP) values equal to or exceeding 130 mmHg and/or diastolic blood pressure (DBP) greater than 80 mmHg [1].

High blood pressure is among the principal risk elements for the emergence of various cardiovascular ailments, including atrial fibrillation, congestive heart failure, coronary artery disease, cerebrovascular disease, peripheral arterial disease, aortic aneurysm, and chronic kidney disease [2].

Indapamide is a thiazide-like diuretic for the management of mild to moderate hypertension as well as relieving symptoms of fluid overload in decompensated heart failure [3]. It blocks the Na^+^/Cl^−^ co-transporter [4] in the proximal and distal tubules. However, inhibition of carbonic anhydrase in the kidney indirectly inhibits sodium/hydrogen exchange and decreases sodium chloride co-transport [5].

Indapamide is available as an immediate-release (IR) 2.5 mg oral formulation, and its antihypertensive activity is related to its vascular properties [6]. Pharmacokinetics [7], metabolism [8], disposition [9], and clinical pharmacology [10] for indapamide immediate-release formulation have been widely studied, while those concerning sustained release were very limited, if any.

Sustained-release antihypertensive medications are more convenient for patients to administer, resulting in a significant improvement of their compliance as well as providing a more consistent blood pressure control by releasing the medication over a longer period of time and preserving blood pressure levels in a narrower range with a reduction in the risk of complications associated with hypertension as heart attack, stroke, and kidney disease.

A sustained-release (SR) dosage form of indapamide was developed at a low dose of 1.5 mg, which was designed to reduce the peak concentrations observed after the administration of repeated immediate-release dosage without a significant reduction in the antihypertensive activity over a period of 24 h with a decrease in the incidence of side effects. The antihypertensive effect and tolerance profile of the SR formulation has been tested by measuring blood pressure and serum potassium levels 24 h after the last drug intake in comparative clinical studies from 2.5 mg IR to 1.5 mg SR [11].

Asmar et al. [11] demonstrated better safety upon administration of low-dose SR 1.5 mg indapamide with the same antihypertensive efficacy of 2.5 mg IR and more than a 50% reduction in the number of individuals presented with serum potassium below 3.4 mmol/L. Additionally, clinical safety data assessed the absence of indapamide effect on carbohydrate and lipid metabolism, indicating an improved safety upon administration of low dose SR 1.5 mg indapamide with the same antihypertensive efficacy [12].

It is worth mentioning that the incidence of hypokalemia is reduced by using a sustained-release formulation of indapamide [13].

Regarding the pharmacokinetics characteristics, the fraction of indapamide released is rapidly absorbed via the gastrointestinal digestive tract. Ingestion of indapamide with food results in a slight increase in the rate and extent of absorption without a clinical significant effect. Following a single dose of indapamide 1.5 mg SR, peak serum level occurs about 12 h after dose administration, where indapamide shows a wide-body distribution, with high binding to specific sites. Indapamide shows a high binding affinity to RBCs (80%) and, more precisely, to carbonic acid anhydrase (98%) without having any remarkable inhibition on its activity. The binding of indapamide to plasma proteins is 79%, with an elimination half-life ranging from 14 to 24 h (an average of 18 h) [14].

It was reported that, under fed conditions, indapamide blood levels were higher with an earlier T_max_ than those observed under fasting ones, with a significant increase (*p* < 0.01) in C_max_ and AUC with food [3]. This significant increase in the rate and extent of absorption may raise a clinical concern about the incidence of potential long-term side effects such as hypokalemia.

Chlorthalidone and indapamide have been found to reduce cardiovascular events, compared to hydrochlorothiazide alone, which showed no evidence of lowering cardiovascular events [15]. Regarding indapamide safety, an in vitro study showed that indapamide had no considerable genotoxic impact on human lymphocytes [16]. Moreover, long-term indapamide therapy, typically in combination with perindopril, showed indications of benefit in terms of mortality and morbidity [17].

On the other hand, at commonly prescribed dosages, indapamide has been determined to be more efficacious than hydrochlorothiazide [18].

From a pharmacogenomics point of view, a study was conducted to evaluate the rate of polymorphisms of *UGT2B4*, *UGT2B7*, and *UGT2B15* in Caucasians and Asians. The study showed that, for all polymorphisms, the genotype and allele frequencies were considerably different in both populations. Asians were homozygous for common alleles, and the incidence of wild-type alleles is two times higher than that of Caucasians [19].

It is well-known that indapamide is metabolized by glucuronidation [20], indicating the possibility of *UGT2B7* polymorphic effect on indapamide metabolism and consequently its pharmacokinetics and pharmacodynamics properties.

The association between pharmacokinetics (PK), pharmacodynamics (PD), and pharmacogenomics (PG) of antihypertensive medications can help to improve their safety and efficacy. Knowing how fast a drug can be absorbed, how it affects the body, and how genes affect the body’s response to this drug can help to determine the correct dosage, identify potential side effects, and those patients who are more likely to experience side effects or who may need a different dosage respectively [21,22].

This study aimed to investigate the impact of food on both the pharmacokinetics and pharmacodynamics properties of indapamide 1.5 mg SR tablet. Furthermore, to investigate the possibility of correlation between *UGT2B7* genetic polymorphisms and potential changes in either/or both of its pharmacokinetics and pharmacodynamics parameters (i.e., the effect of the peak serum concentration and the area under the serum concentration curve on the average SBP, DBP, and pulse). Additionally, to describe the potential changes in clinical efficacy corresponding to the presence of any pharmacokinetic changes and if there is a clinical significance about safety concerns regarding the administration of indapamide 1.5 mg SR tablet with food.

## 2. Methods

### 2.1. Study Design and Setting

A randomized, parallel, single-blind comparative study for the bioavailability of indapamide 1.5 mg SR in healthy adult volunteers under fasting and fed states with a washout period of two weeks. The study was performed at the clinical site, namely, Drug Research Center (DRC), Cairo, Egypt.

### 2.2. Study Procedures

Following at least 10 h of an overnight fast, 25 healthy subjects were administered a single dose of indapamide 1.5 mg SR tablets with 240 mL of water and continued fasting for about 4 h after dose administration (fasting condition). Another 24 healthy subjects started the recommended high-fat meal 30 min before administration of indapamide 1.5 mg SR tablets which was administered with 240 mL of water 30 min after the start of the meal, and no food was permitted in the first 4 h post-dose (fed condition).

**Figure 1:**
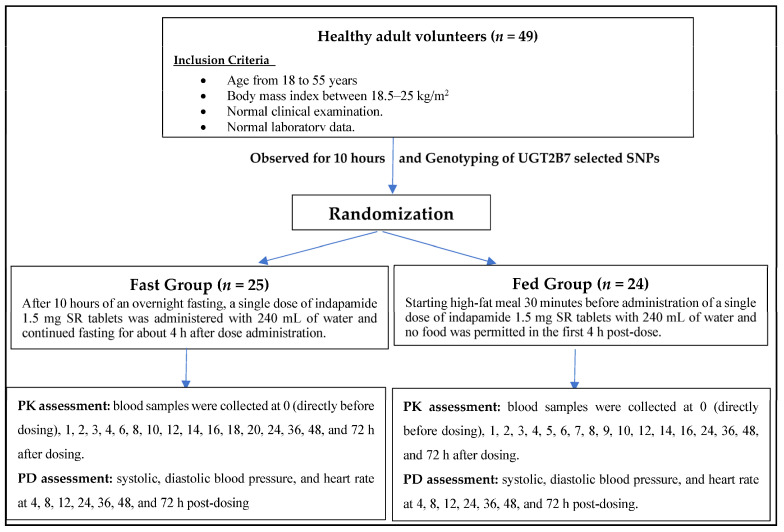
Study Flow Diagram.

Seventeen blood samples were collected from the volunteers at the fasting condition at the following time intervals; 0 (directly before dosing), 1, 2, 3, 4, 6, 8, 10, 12, 14, 16, 18, 20, 24, 36, 48, and 72 h after dosing. On the other hand, eighteen blood samples were collected for the fed state at 0 (directly before dosing), 1, 2, 3, 4, 5, 6, 7, 8, 9, 10, 12, 14, 16, 24, 36, 48, and 72 h after dosing. The biological matrix (whole blood) was collected in tubes containing EDTA disodium, and the whole blood samples were immediately frozen and stored at −80 °C until analysis time (Figure 1).

### 2.3. Subjects

Subjects who fulfilled the inclusion criteria were admitted to the study premises and observed for at least 10 h before dose administration and until collecting the 72 h blood sample.

Inclusion criteria were age from 18 to 55 years, body mass index between 18.5 and 25 kg/m^2^, normal clinical examination, and normal laboratory data. The exclusion criteria were as follows: subjects who administered any medication within less than two weeks of the study starting date. Those who had donated blood and/or participated in clinical studies that required withdrawing more than 500 mL of blood 45 days preceding the study starting date. A history of alcoholism, drug abuse, or smoking more than eight cigarettes per day. A documented drug hypersensitivity or significant medical comorbidities or chronic illnesses including, but not limited to, chronic kidney or liver diseases, cardiovascular system diseases, and diabetes.

### 2.4. Bioanalytical Method Validation

Peak area ratios of varying amounts of indapamide in whole blood in the required concentration range should be highly linear (r^2^ of not less than 0.998). The results of intraday precision C.V. % should be per the latest FDA Guidelines [23]. Accuracy and precision were assessed at three different concentrations in the range of predicted drug concentrations on a within and between-day basis. The lower limit of quantitation must show an adequate quantitation limit to cover small drug concentration ranges during the elimination phase. The drug should demonstrate adequate stability in blood in the studied conditions.

### 2.5. DNA Extraction and Genotyping

Concerning DNA extraction and genotyping of *UGT2B7* selected SNPs, 5 mL blood samples were collected from each participant in EDTA vacutainers before drug administration. The DNA extraction was performed using the illustraTM blood genomicPrep Mini Spin Kit (GE Healthcare UK Limited, Amersham, UK). The concentration of the extracted genomic DNA was measured using the NanoDrop™ ND-1000 Spectrophotometer (Thermo Fisher Scientific, Waltham, MA, USA). The gDNA isolates were all used undiluted in the SNP analysis.

Genotyping of *UGT2B7 rs7438135* and *rs11740316* was performed using TaqMan™ predesigned probes (Thermo Fisher Scientific, Waltham, MA, USA) and the Rotor-Gene QTM real-time PCR instrument (QIAGEN, Hilden, Germany). The reaction plate was prepared using TaqMan™ GTXpress™ Master Mix (Thermo Fisher Scientific, Walthman, MA, USA), gDNA, and RNAse-free water. The thermal profile was as follows: denaturation of the DNA strand at 95 °C for 20 s, hybridization of the primers and probes at 92 °C for 40 s, then elongation at 60 °C for 30 s.

### 2.6. Evaluation of Safety and Tolerability

Assessment of the safety and tolerability of indapamide was performed through monitoring of the incidence of side effects and/or adverse events among the participants during the study. For safety reasons, potassium level was measured pre and post-dose. Moreover, vital signs, including systolic and diastolic blood pressure and heart rate, were measured pre-dosing and after drug intake at 4, 8, 12, 24, 36, 48, and 72 h during the study period. Additionally, the presence of any adverse events, such as dizziness, headache, fatigue, muscle cramps, and gastrointestinal disturbances, were reported during the study.

### 2.7. Ethical Considerations

The current study was conducted as per the ICH-GCP guidelines and the study protocol was approved by Ethics Committee of Faculty of Pharmacy, Ain Shams University under No. 269. The study was registered at ClinicalTrials.gov with Identifier: NCT05294484.

All study aspects were discussed with participants, and written informed consent was signed by the participant and the principal investigator before the commencement of the study. All aspects of the study followed the ethical standards set by the Declaration of Helsinki.

Licensed physicians underwent complete physical examinations and obtained a comprehensive medical history from each participant; in addition to monitoring subjects for the incidence of any adverse drug events, measurement of vital signs such as blood pressure, pulse rate, body temperature, and respiratory rate was performed before and at a specified time during the study course. Finally, registered nurses were responsible for blood sampling.

### 2.8. Statistical Analysis

Regarding pharmacokinetics and pharmacodynamics, the data were presented as mean ± standard deviation for the evaluation of food effect on the pharmacokinetics and pharmacodynamics profiles of indapamide.

For pharmacodynamic parameters, systolic blood pressure (SBP), diastolic blood pressure (DBP), mean arterial blood pressure (MAP), and pulse rate (P) were subjected to statistical analysis using the SPSS program, applying (factorial repeated measures and COVA model). Additionally, pharmacokinetic parameters C_max_, T_max_, AUC, T_1/2_, and MRT_0-inf_ were subjected to multivariate analysis of covariance (Man COVA model). Indapamide pharmacodynamic and pharmacokinetic parameters between the fasting and fed groups were considered statistically significantly different on comparison using the probability of the null hypothesis at a *p*-value of <0.05.

Regarding the genetic data, statistical analysis was performed in R software version 4.0.1 (R Foundation for Statistical Computing, Vienna, Austria). *p*-values less than 0.05 were considered statistically significant results. The Shapiro–Wilk test was performed to check the normality of the data. Parametric data were compared using the Student’s *t*-test or one-way ANOVA, and non-parametric data were compared using the Kruskal–Wallis test or Mann–Whitney’s U test. Multiple linear regression analysis was performed to study the impact of *rs7438135*, *rs11740316*, and food on average systolic and diastolic blood pressures and pulse while adjusting for baseline values. Multiple linear regression analysis was also performed to study the effect of food and the aforementioned SNPs on the pharmacokinetic parameters while adjusting for age and body mass index.

Observed genotype frequencies were checked to be in concordance with the Hardy–Weinberg equilibrium (HWE) by the chi-squared test. Genetic information including both allele and genotype frequencies together with polymorphism information content (heterozgyosity and Hardy–Weinberg equilibrium) were calculated using the genetics package in R software version 4.0.1. In the analysis, all genetic models (dominant, over-dominant, additive, and recessive) were considered.

### 2.9. Sample Size Calculation

The sample size was calculated based on Schuirman’s two-sided *t*-tests. The desired power was 80%, and the type-1 error rate was 5%. Data from a study by Schiavi et al. showed that intra-subject CV% of indapamide under fed conditions is 19.2% [3]. Calculation according to these values produces a minimal sample size of 16 subjects. Assuming a drop-out rate of 20%, the work would be expected to be performed on 50 subjects (screening of about 60 subjects was performed to ensure at least participation of 16 in the study).

## 3. Results

### 3.1. Bioanalytical Method Validation

The bioanalytical method was validated following the US FDA Validation [23]. The validation parameters performed include accuracy, precision, specificity, linearity, matrix effect and recovery, dilution integrity, incurred sample reanalysis, and stability [23]. Whole blood sampling was selected as per FDA requirements [24] as it is highly bound to red blood cells (80%) [13]. The calibration curves were reliable, reproducible, and linear for a concentration range of 0.25–50 ng/mL during the 3-day validation period, and the regression coefficient was always greater than 0.999 for the analyte. The accuracy and precision were always within acceptable ranges of 20% for LLOQ and 15% for low, medium, and high-quality control samples.

### 3.2. Demographic Data

Forty-nine healthy subjects were enrolled in this study. The median patient’s age was 33 years (range: from 18 to 54 years). Both groups (fasting and fed) were matched to age and BMI. However, fed patients had significantly lower blood pressure than fasting patients. There was no remarkable difference in baseline heart rate among groups (Table 1).

### 3.3. Subjects’ Genetic

Both studied SNPs were in concordance with the Hardy–Weinberg equilibrium and were informative. The observed allele and genotype frequencies, polymorphism information content, heterozygosity, and HWE statistics are shown in Table 2.

#### 3.3.1. Bivariate Analysis

Concerning rs7438135, there were no significant differences between the pharmacokinetic parameters across the three genotypes (Table 3). As for *rs11740316*, patients harboring the AG genotype tended to have faster indapamide clearance, shorter drug half-life, and mean residence time (Table 4).

**Figure 2:**
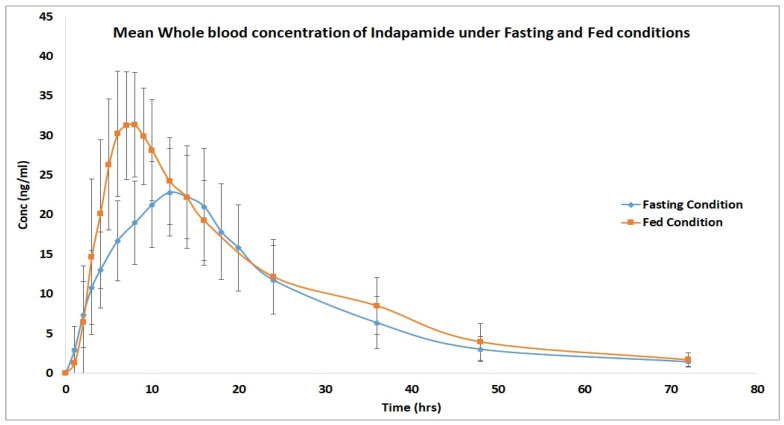
Whole blood concentration (Mean ± SD) of indapamide after administration of indapamide 1.5 mg sustained release film-coated tablets under fasting and fed conditions.

Concerning the impact of food on the pharmacokinetic parameters, food was correlated with lower T_max_, higher C_max_, and higher AUC_0–t_ (Figure 2). Food had no significant impact on average SBP, DBP, and pulse rate (Table 5).

#### 3.3.2. Multiple Linear Regression Analysis

In the additive genetic model (Table 6), all genetic variants in both SNPs had no significant impact on T_max_, C_max_, and AUC_0–t_. However, *rs11740316* variant AG correlated with a 2.8 h decrease in MRT_inf_. In addition, food independently decreased T_max_ by 5.5 h and increased C_max_ by 8.7 ng/mL. Furthermore, older age was associated with a modest increase in AUC_0–t_ and C_max_. Finally, higher BMI correlated with longer MRT_inf_. Both SNPs had no significant impact on indapamide elimination (Table 7).

In addition to the above data, multiple regression models predicting the impact of rs7438135, rs11740316, and food on indapamide pharmacokinetic parameters (using Dominant and Recessive genetic models),as well as, the impact of rs7438135, rs11740316 on indapamide elimination (using Dominant and Recessive genetic models) were presented in Tables S1–S4, respectively. Moreover, Tables S5 and S6 showed the impact of food, rs7438135, rs11740316 on indapamide pharmacodynamic (Dominant and Recessive genetic models respectively).

### 3.4. Pharmacodynamics Parameters

From the data presented in Table 8, it is clear that neither *rs7438135*, *rs11740316*, nor food had a significant impact on the average SBP, DBP, and pulse rate of the participants when adjusted to the effect of baseline SBP, DBP, and pulse.

### 3.5. Evaluation of Safety and Tolerability

A summary of the side effects and/or the adverse events monitored during the study periods after administration of 1.5 mg SR indapamide is given in (Table 9).

A total of 26 treatment-emergent adverse events were reported by 12 subjects out of 49 (12 under fasted conditions and 14 under fed conditions). All the reported adverse events were of mild intensity, and all subjects recovered without sequelae. The most frequently reported biochemical side effect was hypokalemia, which was expected considering it is widely reported after indapamide administration in therapy. However, indapamide was generally well tolerated by subjects without severe adverse events leading to withdrawal from the study.

## 4. Discussion

It is worth mentioning that the clinical importance of indapamide emerged from being an antihypertensive agent for the management of mild to moderately elevated blood pressure [3]. Additionally, it has the advantage of offering an equivalent clinical effect similar to amlodipine 5 mg and hydrochlorothiazide 25 mg, imposing it as an alternative therapy for use in elderly hypertensive patients [25].

On the other hand, a study showed that indapamide significantly outperforms canagliflozin in terms of HbA1c% reduction, insulin receptor substrate 1 (IRS1) expression, and reductions in NF-B and CD68 expression, [26] providing the superiority of indapamide use in hypertensive and type 2 diabetic patients over other antihypertensive agents.

In addition, there is a significant risk reduction for all-cause mortality, cardiovascular death, and fatal stroke; all strokes were reported in indapamide compared to placebo, showing benefits in terms of mortality and morbidity [17].

The main target of formulating indapamide in the sustained release form (SR) was to allow indapamide administration in the lowest possible dose (1.5 mg), giving an advantage of a reduction in peak drug concentrations without a significant reduction in clinical drug activity over the 24 h period, and decreasing the incidence of side effects that may occur [10].

Although it was mentioned in the published literature that there is no clinical significance resulting from the food effect [13], there are significant pharmacokinetic changes [3] that, in turn, may raise safety concerns about the incidence of side effects. Published studies concerning the food effect on indapamide SR tablet pharmacokinetics and dynamics are scarce; therefore, in this regard, the present food effect study was conducted to add to and emphasize the literature data.

In the present study, under fasted conditions, the obtained value of C_max_ was 24.348 ± 6.284 ng/mL with a T_max_ value of 13.600 ± 1.633 h. compared to a higher recorded value of C_max_ (33.827 ± 6.644 ng/mL) with an earlier T_max_ of 8.125 ± 1.895 h under the fed condition. The previously obtained results were in agreement with those reported by Schiavi et al., 2000 [3] for a T_max_ value of 12.3 ± 4.0 h and C_max_ of 26 ± 10 ng/mL under fasting conditions. Compared to the T_max_ value of 9.8 ± 2.3 h and C_max_ of 34 ± 12 ng/mL under fed conditions. Moreover, the public assessment for indapamide reported that food lowered the value of T_max_ for indapamide [27]

It is worth mentioning that the current fed study condition was conducted following a standardized high-fat meal according to that reported by the FDA [28]. From the pharmacodynamics aspect, the results of the current study showed a slightly non-significant difference between the fast and fed groups indicating the absence of clinical effect of food on the pharmacodynamics of indapamide in compliance with what was reported in the literature regarding the probable food effect on indapamide clinical efficacy [13].

The observed difference in the pharmacokinetic profiles of the fasting condition compared to the fed condition showed the absence of any alteration in indapamide 1.5 mg SR tablet clinical efficacy evidenced by undetected abnormal values of the measured blood pressure and heart rate during the study, although the release rate was different.

Regarding the incidence of side and/or adverse effects, no serious effects were observed on the participants in both fast and fed conditions. Although the difference in the C_max_ value was significant, it seems that this difference might not induce any significant concern regarding the safety and efficacy of indapamide 1.5 mg SR tablet upon administration with food. Our study findings showed a total of 26 treatment-emergent side effects that were reported by 12 subjects out of 49, where the most frequent one was hypokalemia, in agreement with that mentioned in the indapamide summary of product characteristics (SmPCs) [29].

It is well-known that one of the metabolic routes of indapamide is glucuronidation [20]. Thus, the current study aimed to investigate the effect of genetic polymorphism via glucuronidation pathway on the pharmacokinetics and pharmacodynamics behavior of a single oral dose of indapamide given to healthy volunteers. Our findings showed that *UGT2B7* polymorphism SNPs *rs7438135* and *rs11740316* had a non-significant effect on indapamide’s pharmacokinetics parameters, thus, paving the road for future studies to be performed on hypertensive patients on indapamide treatment for the long-term which might result in a significant effect.

It is worth mentioning that some dietary substances may interact with indapamide UGT metabolic fate. For instance, garlic boosts UGT expression, potentially decreasing indapamide therapeutic efficacy. On the other hand, turmeric suppresses the expression of UGT enzymes in the liver, which may result in hyponatremia and hypokalemia [30].

The results of the statistical analysis of our study coincided with those recorded in the public assessment report of indapamide [16]. The results of SNPs *rs7438135*, and *rs11740316*, indicated a non-significant difference between the pharmacokinetic parameters of indapamide across the three tested genotypes *GG* (*wild-type*), *AG* (*Heterozygous*), and *AA* (*Homozygous*). Furthermore, SNPs *rs7438135* and *rs11740316* showed a non-significant impact on the average values of the pharmacodynamic parameters in any of the genetic models.

The side effects reported by the participants in the present study were all mild and tolerable. Twenty-six subjects out of forty-nine (twelve under fasted condition and fourteen under fed condition) showed different indapamide-related mild side effects. The reported side effects coincided with what was mentioned in the literature [31].

The current study has certain limitations, including the small sample size, which, if performed on a larger number of volunteers, might show significance in other parameters, including pharmacodynamics as well as genomics.

Moreover, this study was limited to healthy volunteers, being clinical trial phase I, and the obtained results were then restricted to the ideal case of humans with a lack of variabilities included in patients suffering from hypertension as well as those who might have complications from hypertension leading to comorbidity cases. This might result in different results from the current study, either in the significance of more parameters or the absence of significance in other parameters. For that, the obtained results could not be applied typically to hypertensive patients, and further studies are recommended to be performed in the future on hypertensive patients and others suffering from comorbidities.

## 5. Conclusions

The pharmacokinetics of indapamide showed a significant difference in both C_max_ and T_max_ values upon co-administration with food. Results showed that food independently decreased the value of indapamide’s T_max_ by 5.5 h and increased the value of C_max_ by 8.7 ng/mL with unremarkable differences in safety and efficacy.

On the other hand, all genetic variants of both *UGT2B7* SNPs showed a non-significant impact on the values of T_max_, C_max_, and AUC_0–t_; however, it was found that *rs11740316* variant *AG* was correlated with a 2.8-h lower MRT_inf_. Moreover, the participant’s body mass index was positively correlated with longer MRT_inf_.

It was concluded that none of *rs7438135*, *rs11740316*, or food had a significant impact on the pharmacodynamic properties of indapamide in healthy volunteers, although food showed a significant impact on indapamide C_max_ and T_max_ values.

The study findings can assume that indapamide SR tablet administration under fast and fed (high-fat meal) conditions was considered safe and tolerable.

## 6. Recommendations

Further clinical investigation is recommended for assessment of the change in the clinical effect and the incidence of side effects upon co-administration of indapamide SR tablet with different food regimens compared to fasting state after a single and multiple-dose administration. Additionally, it is advisable to consider other dietary habits than a high-fat meal in future studies. Till the previous recommendations are performed, it is advisable to administer indapamide on an empty stomach.

It is recommended that this study be performed on a larger scale as well as on hypertensive patients in order to assess the effect of food and *UGT2B7* genetic polymorphism on the pharmacokinetics and pharmacodynamics of indapamide.

## 7. Limitations

The present study has some limitations, including the following:

- The fed condition arm of the study was a high-fat meal only, while other meals such as low-fat meals, fruits, and juices such as cranberry and grapefruit were not addressed;
- Being performed on healthy volunteers as clinical trial phase 1, the current study lacks the inclusion of hypertensive patients with comorbidities; thus, the results of this study could not be extrapolated to patients with hypertension or other health conditions;
- Further studies on a larger sample size are required.

## Figures and Tables

**Table 1:** Baseline demographics and vital signs of participants for each group.

Demographic Data and Vital Signs	Fasting Condition (*n* = 25)	Fed Condition (*n* = 24)	Total (*n* = 49)	*p*
Age (years):				
Median (Range)	30.0 (18.0–43.0)	38.5 (21.0–54.0)	33.0 (18.0–54.0)	0.07 ^1^
Gender (Male/Female)	22 (males)/3 (females)	Males	46 (males)/3 (females)	
Body weight (kg):				
Mean (SD)	70.2 (9.4)	77.8 (10.3)	72.6 (13.6)	
BMI (kg/m^2^):				0.67 ^2^
Mean (SD)	25.0 (2.8)	25.4 (3.1)	25.2 (2.9)	
Baseline SBP (mmHg):				0.02 ^1^
Median (Range)	120.0 (110.0–130.0)	120.0 (110.0–120.0)	120.0 (110.0–130.0)	
Baseline DBP (mmHg):				<0.01 ^1^
Median (Range)	80.0 (70.0–85.0)	77.5 (70.0–80.0)	80.0 (70.0–85.0)	
Baseline heart rate (bpm):				0.66 ^1^
Median (Range)	72.0 (70.0–81.0)	73.5 (70.0–77.0)	73.0 (70.0–81.0)	

Abbreviations: BMI: Body Mass Index; SBP: Systolic blood pressure; DBP: Diastolic blood pressure; SD: Standard Deviation; ^1^ Mann–Whitney’s U test; ^2^ Student’s *t*-test.

**Table 2:** Genetic information of *rs7438135* and *rs11740316* in the studied groups.

Genetic Information	*rs7438135*	*rs11740316*
Allele frequency:		
* ‘G’*	55 (0.56)	62 (0.63)
* ‘A’*	43 (0.44)	36 (0.37)
Genotype frequency:		
* GG* (*wild-type*)	13 (0.27)	17 (0.35)
* AG* (*heterozygous*)	29 (0.59)	28 (0.57)
* AA* (*homozygous*)	7 (0.14)	4 (0.08)
PIC:	0.37	0.36
Heterozygosity:	0.50	0.47
HWE:		
* *χ^2^	1.43	1.88
* * *p*	0.24	0.17

Abbreviations: HWE, Hardy–Weinberg equilibrium; PIC, Polymorphism information content.

**Table 3:** Comparison between the pharmacokinetic parameters per *rs7438135* genotypes.

		*rs7438135*		
Pharmacokinetic Parameters	*AA* (*n* = 7)	*AG* (*n* = 29)	*GG* (*n* = 13)	*p*
K_e_ (h^−1^)				0.66 ^1^
* *Mean (SD)	0.045 (0.011)	0.048 (0.006)	0.048 (0.006)	
T_max_ (h)				0.78 ^2^
* *Median (Range)	12.0 (6.0–16.0)	12.0 (6.0–16.0)	10.0 (6.0–16.0)	
C_max_				0.99 ^1^
* *Mean (SD)	29.4 (7.0)	29.0 (7.7)	28.8 (9.6)	
AUC_0–t_				0.75 ^2^
* *Median (Range)	687.0 (470.4–955.0)	636.7 (269.8–1346.3)	714.4 (282.8–884.0)	
T_0.5_ (h)				0.62 ^2^
* *Median (Range)	15.0 (12.6–17.2)	13.9 (11.9–21.4)	15.7 (11.1–24.6)	
MRT_inf_				0.74 ^1^
* *Mean (SD)	24.7 (2.5)	24.5 (3.1)	25.5 (5.1)	

^1^ One-way ANOVA; ^2^ Kruskal–Wallis rank-sum test.

**Table 4:** Comparison between the pharmacokinetic parameters per *rs11740316* genotypes.

		*rs11740316*		
Pharmacokinetic Parameters	*GG* (*n* = 17)	*AG* (*n* = 28)	*AA* (*n* = 4)	*p*
K_e_ (h^−1^)				0.13 ^1^
* *Mean (SD)	0.046 (0.007)	0.049 (0.008)	0.042 (0.007)	
T_max_ (h)				0.92 ^2^
* *Median (Range)	12.0 (6.0–16.0)	12.0 (6.0–16.0)	12.0 (6.0–16.0)	
C_max_				0.59 ^2^
* *Median (Range)	28.0 (15.6–41.5)	28.8 (12.0–43.3)	29.7 (28.9–41.8)	
AUC_0–t_				0.94 ^1^
* *Mean (SD)	659.5 (175.6)	676.0 (223.1)	694.3 (79.6)	
T_0.5_ (h)				0.08 ^2^
* *Median (Range)	15.2 (12.1–22.5)	13.7 (11.1–24.6)	16.8 (13.8–20.7)	
MRT_inf_				0.14 ^1^
* *Mean (SD)	26.1 (3.9)	23.9 (3.3)	(3.5)	

^1^ One-way ANOVA; ^2^ Kruskal–Wallis rank-sum test.

**Table 5:** Comparison between pharmacokinetic and pharmacodynamic parameters in fasting and fed subjects.

PK/PD Parameters	Fasting (*n* = 25)	Fed (*n* = 24)	*p*
T_max_			<0.01 ^1^
* *Median (Range)	14.0 (12.0–16.0)	8.0 (6.0–14.0)	
C_max_			<0.01 ^2^
* *Mean (SD)	24.3 (6.3)	33.8 (6.6)	
AUC_0–t_			0.02 ^2^
* *Mean (SD)	610.3 (165.0)	735.8 (210.0)	
MRT_inf_			0.41 ^2^
* *Mean (SD)	25.2 (3.5)	24.4 (3.8)	
Average SBP:			0.43 ^1^
* *Median (Range)	113.3 (106.7–118.3)	113.3 (106.7–116.7)	
Average DBP:			0.20 ^1^
* *Median (Range)	71.7 (70.0–75.0)	70.0 (65.0–76.7)	
Average pulse:			0.58 ^2^
* *Median (Range)	74.3 (70.3–78.3)	75.0 (71.3–77.7)	

Abbreviations: PK/PD: Pharmacokinetics/Pharmacodynamics; SBP: Systolic blood pressure; DBP: Diastolic blood pressure; SD: Standard Deviation; ^1^ One-way ANOVA; ^2^ Kruskal–Wallis rank-sum test.

**Table 6:** Multiple regression models predicting the impact of *rs7438135*, *rs11740316*, and food on indapamide pharmacokinetic parameters.

Study Condition and Genetic Information	T_max_	C_max_	AUC_0–t_	MRT_inf_
	Estimates	Estimates	Estimates	Estimates
(Intercept)	14.6 *** (9.7–19.5)	31.9 *** (15.3–48.5)	422.9 (−67.0–912.7)	16.2 *** (7.1–25.3)
Age (years):	0.004 (−0.07–0.07)	0.3 * (0.02–0.49)	8.3 * (1.4–15.2)	0.0 (−0.1–0.1)
BMI (kg/m^2^):	−0.1 (−0.3–0.2)	−0.7 (−1.4–0.1)	−1.7 (−23.9–20.5)	0.5 * (0.04–0.86)
Food Effect:				
* *Fasting	Reference	Reference	Reference	Reference
* *Fed	−5.5 *** (−6.7–−4.4)	8.7 *** (4.8–12.5)	82.0 (−32.5–196.5)	−1.2 (−3.3–1.0)
*rs7438135:*				
* GG* (*wild-type*)	Reference	Reference	Reference	Reference
* AG* (*Heterozygous*)	0.1 (−1.2–1.4)	−0.4 (−4.8–4.0)	−25.8 (−155.5–103.8)	−0.6 (−3.0–1.8)
* AA* (*Homozygous*)	−0.1 (−1.9–1.8)	3.0 (−3.3–9.4)	−12.0 (−200.0–175.9)	−2.1 (−5.6–1.4)
*rs11740316:*				
* GG* (*wild-type*)	Reference	Reference	Reference	Reference
* AG* (*Heterozygous*)	0.4 (−0.8–1.7)	1.6 (−2.5–5.6)	1.3 (−119.3–122.0)	−2.8 * (−5.0–−0.5)
* AA* (*Homozygous*)	1.1 (−1.2–3.3)	4.9 (−2.6–12.4)	−26.8 (−248.5–194.8)	−2.2 (−6.3–1.9)

Abbreviations: BMI: Body Mass Index. * *p* < 0.05, *** *p* < 0.001.

**Table 7:** Multiple regression models predicting the impact of *rs7438135* and *rs11740316* on indapamide elimination.

Study Condition and Genetic Information	T_0.5_	Ke
Predictors	Estimates	Estimates
(Intercept)	14.4 *** (6.77–21.9)	0.047 *** (0.027–0.068)
Age (years):	0.03155 (−0.07–0.13)	−0.00012 (−0.00039–0.00016)
BMI (kg/m^2^):	0.05 (−0.30–0.39)	0.00001 (−0.00091–0.00093)
*rs7438135:*		
* GG* (*wild-type*)	Reference	Reference
* AG* (*Heterozygous*)	−1.16 (−3.17–0.84)	0.00179 (−0.00359–0.00718)
* AA* (*Homozygous*)	−1.58 (−4.43–1.27)	0.00275 (−0.00489–0.01039)
*rs11740316:*		
* GG* (*wild-type*)	Reference	Reference
* AG* (*Heterozygous*)	−0.95 (−2.82–0.93)	0.00339 (−0.00164–0.00841)
* AA* (*Homozygous*)	0.80 (−2.63–4.23)	−0.00255 (−0.01175–0.00665)

Abbreviations: BMI: Body Mass Index. *** *p* < 0.001.

**Table 8:** Impact of food, *rs7438135*, *rs11740316* on indapamide pharmacodynamics (additive model).

Study Condition and Genetic Information	SBP	DBP	Pulse
Predictors	Estimates	Estimates	Estimates
(Intercept)	83.6 *** (61.9–105.3)	47.5 *** (30.5–64.5)	59.3 *** (44.6–74.1)
Baseline	0.2 * (0.0–0.4)	0.3 ** (0.1–0.5)	0.2 * (0.0–0.4)
Food:			
* *Fasting	Reference	Reference	Reference
* *Fed	0.1 (−1.8–1.9)	0.3 (−1.4–2.0)	0.3 (−0.8–1.3)
*rs7438135:*			
* GG*	Reference	Reference	Reference
* AG*	1.1 (−1.0–3.1)	0.3 (−1.4–2.0)	−0.6 (−1.9–0.6)
* AA*	1.7 (−1.3–4.6)	0.3 (−2.1–2.8)	−0.5 (−2.3–1.3)
*rs11740316:*			
* GG*	Reference	Reference	Reference
* AG*	0.5 (−1.4–2.5)	0.8 (−0.8–2.4)	0.1 (−1.1–1.2)
* AA*	2.7 (−0.7–6.2)	2.7 (−0.2–5.6)	0.5 (−1.6–2.5)

Abbreviations: SBP: Systolic blood pressure; DBP: Diastolic blood pressure. * *p* < 0.05, ** *p* < 0.01, *** *p* < 0.001.

**Table 9:** Summary of the reported adverse events after administration of 1.5 mg SR tablets indapamide.

Reported Adverse Events after Administration of Indapamide 1.5 mg SR Tablets			
Fasting State (*n* = 12)		Fed State (*n* = 14)	
Dizziness	0	Dizziness	1
Headache	3	Headache	4
Fatigue	3	Fatigue	2
Muscle cramps	1	Muscle cramps	2
Gastrointestinal disturbances	1	Gastrointestinal disturbances	0
Hypokalemia	4	Hypokalemia	5

*n*: number of adverse events.

## Supplementary Materials

The following supporting information can be downloaded at: https://www.mdpi.com/article/10.3390/biomedicines11051501/s1, Table S1: Multiple regression models predicting the impact of *rs7438135*, *rs11740316*, and food on indapamide pharmacokinetic parameters (dominant genetic model); Table S2: Multiple regression models predicting the impact of *rs7438135* and *rs11740316* on indapamide elimination (dominant genetic model); Table S3: Multiple regression models predicting the impact of *rs7438135*, *rs11740316*, and food on indapamide pharmacokinetic parameters (recessive genetic model); Table S4: Multiple regression models predicting the impact of *rs7438135* and *rs11740316* on indapamide elimination (recessive genetic model); Table S5: Impact of food, *rs7438135*, *rs11740316* on indapamide pharmacodynamics (Dominant model); Table S6: Impact of food, *rs7438135*, *rs11740316* on indapamide pharmacodynamics (Recessive model).
