# Supplementary material for: Association between Food/UGT2B7 Polymorphisms and Pharmacokinetics/Pharmacodynamics Properties of Indapamide in Healthy Humans
Source: Biomedicines. 2023 May 22;11(5):1501. doi: 10.3390/biomedicines11051501 (PMC10216553; doi:10.3390/biomedicines11051501)
Supplement: Supplementary file 1 [file biomedicines-11-01501-s001.zip › biomedicines-2366136-supplementary.pdf]

Dominant Model:

In the dominant model (**Table S1**), again, all genetic variants in both SNPs had no significant impact on T_{max}, C_{max}, and AUC0-t. However, rs11740316 variant AG was independently correlated with a 2.4 h decrease in MRT inf. In addition, food independently decreased T_{max} by 5.5 h and increased C_{max} by 8.1 ng/ml. Furthermore, older age was associated with a modest increase in C_{max}. Finally, higher BMI correlated with longer MRTinf. Both SNPs had no significant impact on indapamide elimination (**Table S2**).

Table S1. Multiple regression models predicting the impact of rs7438135, rs11740316, and food on indapamide pharmacokinetic parameters (dominant genetic model).

	**T_{max}**	**C_{max}**	**AUC_{0-t}**	**MRT_{inf}**
	**Estimates**	**Estimates**	**Estimates**	**Estimates**
(Intercept)	14.5 *** (9.8–19.3)	29.8 *** (13.5–46.2)	422.0 (−49.3–893.4)	16.7 *** (7.9–25.6)
Age (years):	0.0 (−0.1–0.1)	0.3 * (0.0–0.5)	8.2 * (1.5–14.8)	0.0 (−0.1–0.1)
BMI (kg/m²):	−0.1 (−0.3–0.2)	−0.6 (−1.3–0.2)	−1.5 (−22.9–19.8)	0.4 * (0.0–0.8)
Food effect:				
**Fasting**	Reference	Reference	Reference	Reference
**Fed**	−5.5 *** (−6.6–4.4)	8.1 *** (4.3–12.0)	81.2 (−28.5–191.0)	−1.0 (−3.1–1.1)
rs7438135:				
**GG**	Reference	Reference	Reference	Reference
**AG + AA**	0.0 (−1.2–1.2)	−0.2 (−4.4–4.0)	−19.8 (−140.7–101.1)	−1.0 (−3.2–1.3)
rs11740316:				
**GG**	Reference	Reference	Reference	Reference
**AG + AA**	0.5 (−0.6–1.7)	1.3 (−2.6–5.2)	−4.3 (−116.6–108.0)	−2.4 * (−4.5–0.3)

* $p < 0.05$, *** $p < 0.001$.

Table S2. Multiple regression models predicting the impact of rs7438135 and rs11740316 on indapamide elimination (dominant genetic model).

	**$T_{0.5}$**	**K_e**
**Predictors**	Estimates	Estimates
**(Intercept)**	14.35547 *** (6.76807–21.94286)	0.04743 *** (0.02709–0.06778)
**Age (years):**	0.03155 (−0.07010–0.13321)	−0.00012 (−0.00039–0.00016)
**BMI (kg/m²):**	0.04609 (−0.29786–0.39003)	0.00001 (−0.00091–0.00093)
**rs7438135:**		
GG (wild-type)	Reference	Reference
AG (Heterozygous)	−1.16456 (−3.17243–0.84332)	0.00179 (−0.00359–0.00718)
AA (Homozygous)	−1.58218 (−4.43174–1.26738)	0.00275 (−0.00489–0.01039)
**rs11740316:**		
GG (wild-type)	Reference	Reference
AG (Heterozygous)	−0.94623 (−2.81987–0.92740)	0.00339 (−0.00164–0.00841)
AA (Homozygous)	0.79946 (−2.63325–4.23218)	−0.00255 (−0.01175–0.00665)

*** $p < 0.001$.

Recessive Model:

In the recessive model (**Table S3**), again, all genetic variants in both SNPs had no significant impact on Tmax, Cmax, and AUC0-t. In addition, food independently decreased Tmax by 5.5 h and increased Cmax by 8.6 ng/mL. Furthermore, older age was associated with a modest increase in Cmax and AUC0-t. Both SNPs and other covariates had no significant impact on indapamide elimination kinetics (**Table S4**).

Table S3. Multiple regression models predicting the impact of rs7438135, rs11740316, and food on indapamide pharmacokinetic parameters (recessive genetic model).

	**T_{max}**	**C_{max}**	**$AUC_{0\text{-}t}$**	**MRT_{inf}**
Predictors	Estimates	Estimates	Estimates	Estimates
(Intercept)	14.6 *** (9.8–19.3)	31.2 *** (15.3–47.2)	403.4 (−64.6–871.3)	16.3 ** (7.0–25.7)
Age (years):	0.0 (–0.1–0.1)	0.3 * (0.0–0.5)	8.0 * (1.4–14.6)	–0.0 (–0.1–0.1)
BMI (kg/m²):	–0.0 (–0.3–0.2)	–0.6 (–1.3–0.1)	–1.3 (–22.5–20.0)	0.4 (–0.1–0.8)
Food effect:				
**Fasting**	Reference	Reference	Reference	Reference
**Fed**	–5.5 *** (–6.7–4.4)	8.6 *** (4.9–12.4)	83.9 (–27.6–195.4)	–1.0 (–3.3–1.2)
rs7438135:				
**GG + AG**	Reference	Reference	Reference	Reference
**AA**	–0.3 (–1.9–1.3)	2.9 (–2.4–8.2)	6.9 (–149.2–163.0)	–0.8 (–3.9–2.3)
rs11740316:				
**GG + AG**	Reference	Reference	Reference	Reference
**AA**	0.7 (–1.3–2.7)	3.8 (–2.9–10.5)	–21.2 (–218.4–176.0)	0.0 (–3.9–4.0)

* $p < 0.05$, ** $p < 0.01$, *** $p < 0.001$.

Table S4. Multiple regression models predicting the impact of rs7438135 and rs11740316 on indapamide elimination (recessive genetic model).

	**$T_{0.5}$**	**Ke**
Predictors	Estimates	Estimates
(Intercept)	14.35547 *** (6.76807–21.94286)	0.04743 *** (0.02709–0.06778)
**Age (years):**	0.03155 (–0.07010–0.13321)	–0.00012 (–0.00039–0.00016)
**BMI (kg/m²):**	0.04609 (–0.29786–0.39003)	0.00001 (–0.00091–0.00093)
**rs7438135:**		
GG (wild-type)	Reference	Reference
AG (Heterozygous)	–1.16456 (–3.17243–0.84332)	0.00179 (–0.00359–0.00718)
AA (Homozygous)	–1.58218 (–4.43174–1.26738)	0.00275 (–0.00489–0.01039)
**rs11740316:**		
GG (wild-type)	Reference	Reference
AG (Heterozygous)	–0.94623 (–2.81987–0.92740)	0.00339 (–0.00164–0.00841)
AA (Homozygous)	0.79946 (–2.63325–4.23218)	–0.00255 (–0.01175–0.00665)

*** $p < 0.001$.

Table S5. Impact of food, rs7438135, rs11740316 on indapamide pharmacodynamics (Dominant model).

	**SBP**	**DBP**	**Pulse**
Predictors	Estimates	Estimates	Estimates
(Intercept)	84.5 *** (62.9–106.1)	46.9 *** (30.0–63.8)	58.9 *** (44.6–73.2)
Baseline:	0.2 * (0.0–0.4)	0.3 ** (0.1–0.5)	0.2 * (0.0–0.4)
Food:			
**Fasting**	Reference	Reference	Reference
**Fed**	–0.0 (–1.9–1.8)	0.3 (–1.3–2.0)	0.2 (–0.8–1.3)
rs7438135:			
**GG**	Reference	Reference	Reference
**AG + AA**	1.0 (–1.0–2.9)	0.1 (–1.5–1.7)	–0.6 (–1.8–0.5)
rs11740316:			
**GG**	Reference	Reference	Reference
**AG + AA**	0.7 (–1.1–2.6)	1.0 (–0.5–2.6)	0.1 (–1.0–1.2)

* $p < 0.05$, ** $p < 0.01$, *** $p < 0.001$. SBP: Systolic blood pressure, DBP: Diastolic blood pressure.

Table S6. Impact of food, rs7438135, rs11740316 on indapamide pharmacodynamics (Recessive model).

	**SBP**	**DBP**	**Pulse**
Predictors	Estimates	Estimates	Estimates
(Intercept)	82.3 *** (61.2–103.4)	46.2 *** (30.1–62.3)	59.8 *** (45.3–74.3)
Baseline:	0.2 ** (0.1–0.4)	0.3 ** (0.1–0.5)	0.2 * (0.0–0.4)
Food:			
**Fasting**	Reference	Reference	Reference
**Fed**	0.1 (–1.7–2.0)	0.4 (–1.3–2.1)	0.3 (–0.8–1.3)
rs7438135:			
**GG + AG**	Reference	Reference	Reference
**AA**	0.8 (–1.7–3.3)	0.0 (–2.1–2.1)	–0.1 (–1.6–1.5)
rs11740316:			
**GG + AG**	Reference	Reference	Reference
**AA**	2.2 (–1.0–5.3)	2.0 (–0.6–4.6)	0.6 (–1.4–2.5)

* $p < 0.05$, ** $p < 0.01$, *** $p < 0.001$. SBP: Systolic blood pressure, DBP: Diastolic blood pressure.
